# Hepatitis E virus in wild and domestic rabbits from Portugal: a combined molecular and longitudinal serological study

**DOI:** 10.1007/s11259-024-10452-7

**Published:** 2024-06-27

**Authors:** Sérgio Santos-Silva, Nuno Santos, Pedro López-López, Maria S. J. Nascimento, Helena M. R. Gonçalves, Wim H. M. Van der Poel, António Rivero-Juarez, João R. Mesquita

**Affiliations:** 1 School of Medicine and Biomedical Sciences (ICBAS), University of Porto, Porto, Portugal; 2 CIBIO/InBio, Research Center in Biodiversity and Genetic Resources, Campus of Vairão, University of Porto, Vila do Conde, Portugal; 3 Unit of Infectious Diseases, Hospital Universitario Reina Sofia, Clinical Virology and Zoonoses, Instituto Maimonides de Investigación Biomédica de Córdoba (IMIBIC), Universidad de Córdoba (UCO), Cordoba, Spain; 4 Center for Biomedical Research Network (CIBER) in Infectious Diseases, Health Institute Carlos III, Madrid, Spain; 5 Faculty of Pharmacy, University of Porto (FFUP), Porto, Portugal; 6 LAQV, REQUIMTE, Department of Chemistry and Biochemistry, Faculty of Sciences, University of Porto, Porto, Portugal; 7 Quantitative Veterinary Epidemiology, Wageningen University, Wageningen, The Netherlands; 8 Department Virology & Molecular Biology, Wageningen Bioveterinary Research, Lelystad, the Netherlands; 9 Epidemiology Research Unit (EPIUnit), Public Health Institute of the University of Porto, Porto, Portugal; 10 Laboratory for Integrative and Translational Research in Population Health (ITR), Porto, Portugal

**Keywords:** One health, Lagomorphs, Hepatitis E virus, Zoonoses, Seroprevalence

## Abstract

Hepatitis E virus (HEV), species *Paslahepevirus balayani*, poses a global public health threat, especially in developing countries, by causing acute enterically transmitted hepatitis. HEV infects various mammalian hosts and belongs to the genus *Paslahepevirus* in the family *Hepeviridae*. While swine are recognized as the main hosts of HEV, rabbits, which can also be affected by swine HEV-3 related strains, serve as the primary reservoir for the distinct emerging and zoonotic HEV-3ra subtype. In Portugal, where the European wild rabbit is abundant, their role in HEV epidemiology remains unclear. The primary aim of the present research was to evaluate the circulation and the potential for HEV infection within these species. This study employed a molecular and longitudinal serological approach to investigate HEV in Portuguese rabbits. Among the 205 wild rabbits tested, a seroprevalence of 2.44% (95% CI: 0.80–5.60) was found, with no significant associations with age, sex, localization, or sampling dates. Seropositive animals were found in the south and center regions of the country. HEV RNA was not detected in 120 fecal samples, suggesting a natural, low level, and widespread viral circulation. The study underscores the need for further research to comprehend HEV dynamics in these species, which is crucial for assessing potential transmission risks to humans.

## Introduction

Hepatitis E virus (HEV), species *Paslahepevirus balayani* is the most common cause of acute enterically transmitted hepatitis in developing countries (World Health Organization. WHO 2022), being a rising issue that is increasingly alarming public health globally (Raji et al. 2022).

HEV belongs to the family *Hepeviridae*, genus *Paslahepevirus* with the specific species designation *balayani* (Purdy et al. 2022). This family is additionally divided into the subfamily *Parahepevirinae* and *Orthohepevirinae.* The members of the subfamily *Parahepevirinae* exclusively infect trout and salmon, whereas the members of the subfamily *Orthohepevirinae* affect mammals and birds. The subfamily *Orthohepevirinae* is further subdivided into four genera, *Paslahepevirus*, *Avihepevirus*, *Rocahepevirus*, and *Chirohepevirus*. Among these, the genus *Paslahepevirus* consists of two species, including *P. balayani*, which comprises HEV genotypes capable of infecting humans and other mammalian species (Nishizawa et al. 2021) and *P. alci*, infecting moose. HEV can be categorized into eight distinct genotypes, HEV-1 to HEV-8. Genotypes 1 and 2 are restricted to human infections, whereas genotypes 3, 4, and 7 can infect both humans and animals. Conversely, genotypes 5, 6, and 8 solely infect animals (Smith et al. 2020).

Zoonotic genotypes 3 and 4 are predominantly disseminated by consuming pork and pork-related products from infected animals, or by direct interaction with infected animals, especially pigs (Velavan et al. 2021). Genotype 3 is present in all other parts of the globe and is the primary genotype of HEV detected in Europe (Pallerla et al. 2020; Takahashi et al. 2020).

Although swine are recognized as the main hosts of HEV-3 (Purdy et al. 2022), rabbits, which can also be affected by swine HEV-3 related strains (Hammerschmidt et al. 2017; Parisi et al. 2019), serve as the primary reservoir for the distinct emerging and zoonotic HEV-3ra subtype (Cossaboom et al. 2011). The first detection of HEV-3ra in domestic and wild rabbits was in France in 2012 (Izopet et al. 2012). Since then, HEV RNA and anti-HEV antibodies have been detected in lagomorphs in several European countries, including France (Lhomme et al. 2015), United Kingdom, Italy (Di Bartolo et al. 2016; Parisi et al. 2019), Netherlands (Burt et al. 2016), Poland (Bigoraj et al. 2020) and Germany (Eiden et al. 2016; Hammerschmidt et al. 2017; Ryll et al. 2018; Corman et al. 2019).

In the Iberian Peninsula, the European wild rabbit (*Oryctolagus cuniculus*) is a native species that is locally abundant and of significant hunting interest. These animals are an essential food source for humans, particularly in rural areas, often for personal consumption, given that rabbit meat is highly nutritious (Dalle Zotte and Szendro 2011).

Circulation of HEV-3 in the Iberian Peninsula has been identified in wildlife species, including wild boar (*Sus scrofa*) (Rivero-Juarez et al. 2018; Santos-Silva et al. 2023), red deer (*Cervus elaphus*) (Kukielka et al. 2016; Moraes et al. 2022), and the Iberian lynx (*Lynx pardinus*) (Caballero-Gómez et al. 2022). To date, there have been no studies documenting active HEV infections in rabbits in Portugal. However, there is a report of seropositivity in the southern part of Portugal, which explored HEV exposure in a population of wild lagomorphs (Lopes and Abrantes 2020). Consequently, the potential contribution of these species to the HEV epidemiology in Portugal is still far from being known. Therefore, the aim of the current study was to investigate the role of rabbits as reservoirs for HEV in Portugal and to identify potential risk factors linked to HEV exposure in these animals.

## Materials and methods

### Sampling location

For a comprehensive longitudinal study, sera from rabbits were collected using robust capture-mark-recapture methods (Kendall et al. 1995). The collection was conducted on European rabbits belonging to the southwestern Iberian subspecies *Oryctolagus cuniculus algirus*. The study encompassed various locations in southern Portugal, including two free-ranging populations at Companhia das Lezírias (38°5,094,499 N, 8° 5,194,999 W) and Mértola (37°4,392,799 N, 7°4,093,499 W), as well as fenced populations within four enclosures ranging from 0.3 to 4.7 hectares at Parque Natureza Noudar (38°1,190,499 N, 7°0292499 W) and Companhia das Lezírias (38°5,093,499 N, 8°4,893,099 W). Cross-sectional sampling was also conducted in two additional free-ranging populations, namely, Vale Perditos (37°4,991,899 N, 7°2,294,599 W) and Alpiarça (39°1,592,599 N, 8°3,392,699 W).

The landscape of the study locations featured a mosaic of scrub vegetation, primarily composed of *Cistus* sp., *Lavandula* sp., and *Ulex* sp., with sparse cork oak (*Quercus suber*) forest at Companhia das Lezírias and Alpiarça, and holm oak (*Quercus ilex*) at Parque Natureza Noudar, Mértola, and Vale Perditos. Harvesting occurred in the Mértola and Vale Perditos populations, while the Companhia das Lezírias and Alpiarça populations were left unmanaged. In Mértola and Vale Perditos, natural food was supplemented with cereal, while fenced sites (Parque Natureza Noudar and Companhia das Lezírias) provided water and commercial feed year-round, with predation by terrestrial carnivores prevented by 2-meter-high fences equipped with perimeter electrical wire.

Additionally, the sources of water in these free-ranging populations primarily relied on natural water sources, including streams and ponds, while fenced populations had controlled water supplies.

For free-ranging populations, 30 to 52 cage traps were strategically placed, evenly distributed across an approximately 13-hectare area per location. In fenced sites, 10 to 15 cage traps were positioned near feeders within each enclosure. Traps were set 2 h before sunset, baited with vegetables, and checked 2 h after sunset and again 1 h after sunrise, remaining closed during daylight hours.

### Sampling and data collection

**Fig. 1:**
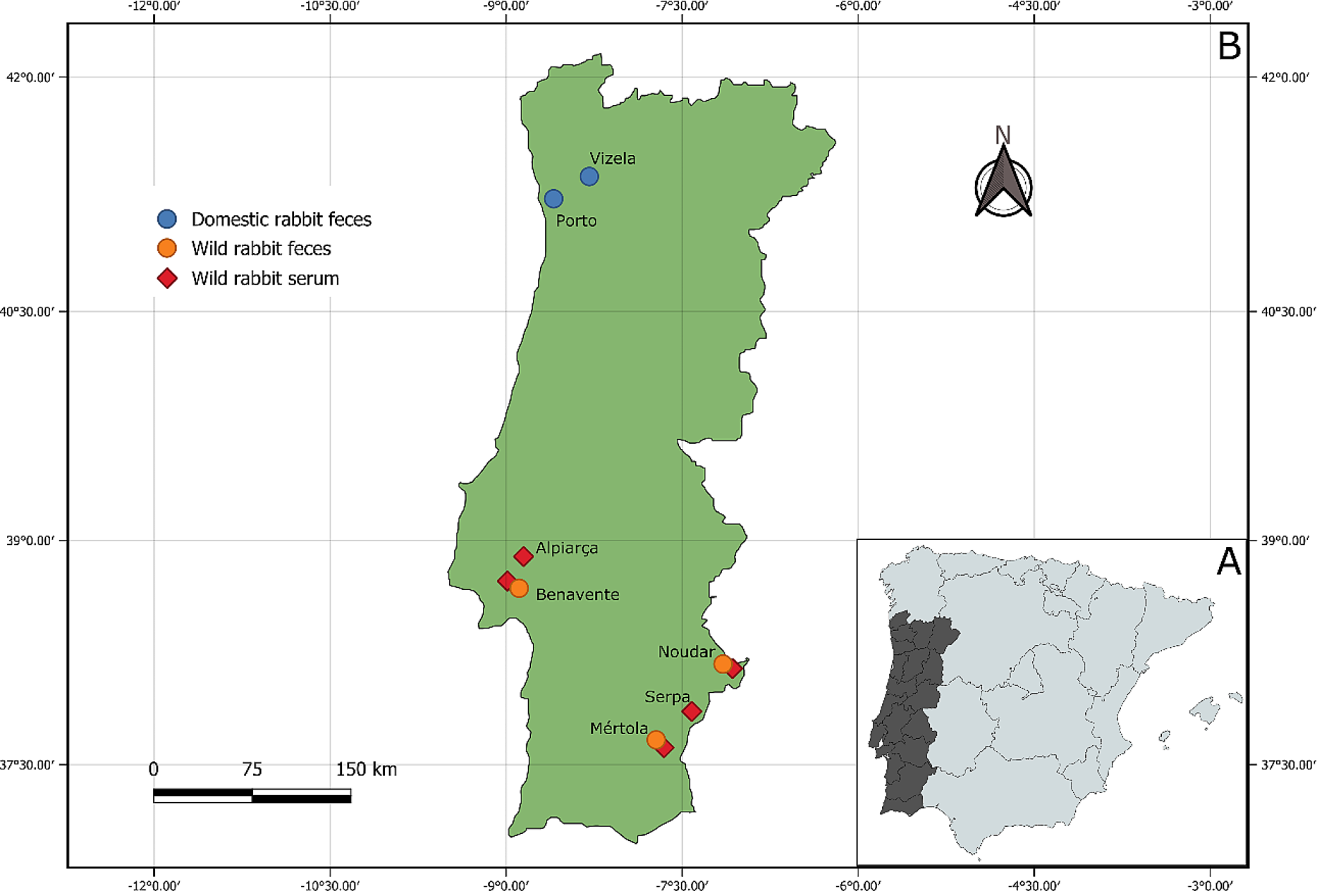
A map of Portugal indicating where rabbit samples were taken (**B**), distinguishing between serum and feces, and wild versus domestic rabbits. Map (**A**) highlights Portugal’s southwest location on the Iberian Peninsula

A total of 205 rabbits were tested for serological markers, more specifically for anti-HEV antibodies. Sera from 165 of these 205 animals were collected on a single occasion. The other 40 rabbits were longitudinally studied by capture-recapture at least one time (in a total of 93 sera). Sera samples were obtained from wild rabbits of two districts from the central and southern regions of Portugal (Fig. 1) between 2018 and 2021.

Each rabbit was individually identified using a subcutaneous microchip upon initial capture. A volume of up to 1.5 mL of whole blood (approximately 0.25% of body weight) was obtained through venipuncture of the saphenous vein and placed in a clotting tube. After centrifugation at 1,430 x *g* for 10 min, the sera were preserved at -20 °C until serological analyses. Sex determination was conducted through visual examination of the external genitalia, while weight was measured with scales (precision of 1 g). Weight demonstrates a strong correlation with age, up to 0.8 kg or 4 months of age, as validated by growth curves (Ferreira and Ferreira 2014). Following processing, rabbits were promptly released at the capture site.

Live trapping and sample collection adhered to permits CIBIO ORBEA/2023_01, ICNF 580/2018/CAPT, 8/2019/CAPT, 197/2020/CAPT, 23/2021/CAPT, and 2-DGVF/DRCA/2021, aligning with European Union directives on the protection of animals used for scientific purposes (Directive 2010/63/EU) and international wildlife standards (Sikes and Gannon 2011).

Additionally, a set of 120 fecal samples from wild and domestic rabbits were also collected for viral detection between 2016 and 2022. Of these, 59 stools were from wild rabbits which blood samples were also collected from located in the southern and central regions of Portugal, and 61 were from domestic pet rabbits from the northern region of Portugal. The fresh feces samples were collected from the floor after the rabbit had defecated. All stool samples were kept at 4º C and transported to the lab within 12 h. Samples were then stored at -20º C until nucleic acid extraction, which was completed within 2 weeks of collection.

### Serological analysis

A commercially available double-antigen sandwich multi-species ELISA kit (HEV 4.0v; MP Diagnostics, Illkirch, France) was used to evaluate the presence of total anti-HEV antibodies. The ELISA was performed following the instructions provided by the manufacturer. This assay is designed based on a highly conserved recombinant protein ET2.1 of the HEV capsid (Hu et al. 2008). It can detect anti-HEV antibodies in serum or plasma of various animal species, including rabbits.

### Nucleic acid extraction

Total nucleic acid was extracted from 200 µL of each individual suspended stool sample. Fecal suspensions (10%) were prepared in phosphate-buffered saline pH 7.2 and centrifuged for 5 min at 8000 × *g*. Extraction was performed using the QIAamp Cador Pathogen Mini Kit (Qiagen, Hilden, Germany), according to the manufacturer’s instructions, in the QIAcube^®^ automated platform (Qiagen). The extraction kit used is proved to have high sensitivity in detecting small amounts of RNA. This kit also efficiently isolates both DNA and RNA, ensuring reliable detection of low viral RNA levels. Eluted total DNA/RNA was stored at -80 ºC with RNase-free water.

### Molecular detection of HEV

To detect HEV, a broad-spectrum nested RT-PCR assay was used targeting the RNA-dependent RNA-polymerase (*RdRp*) gene of the ORF1 region of the HEV genome (amplicon length: 331–334 ) spanning nt 4285–4616 (numbering according to genotype 3 strain Meng accession number AF082843) (Johne et al. 2010). For the first round, Xpert One-Step RT-PCR kit (GriSP^®^, Porto, Portugal) was used for the broad-spectrum nested RT-PCR and for the second round, 5 µL of the first-round products were used as templates with Xpert Fast Hotstart Mastermix 2x with dye (GriSP^®^, Porto, Portugal), all according to the manufacturer’s instructions. The WHO PEI 6329/10 subgenotype 3a standard (accession number AB630970, provided by the Paul Ehrlich-Institute, Langen, Germany) was used as a positive control and RNase-free water as negative control. Amplification reactions, with the corresponding positive and negative controls, were conducted in a Bio-Rad T100TM Thermal Cycler. The amplified fragments were detected by subjecting the PCR amplification products to electrophoresis on 1% agarose gels stained with Xpert Green Safe DNA gel dye (GriSP^®^) at a voltage of 120 V for 30 min. UV light was used to validate and verify the obtained outcomes.

Additionally, HEV RNA detection and quantification was also attempted using a broad-spectrum real-time RT-PCR (RT-qPCR) assay targeting the open reading frame (ORF3) region with primers/probe (TaqMan) as previously described (Jothikumar et al. 2006). The real time PCR reactions were run on a CFX Connect Real-Time thermocycling System (Bio-Rad Laboratories, USA). The RT-qPCR was performed using iTaq Universal Probes One-Step Kit (Bio-Rad Laboratories, USA) at a final total volume of 20 µL reaction mixture in a CFX Connect Real-Time thermocycling System (Bio-Rad Laboratories, USA). For the RT-qPCR, the same positive (WHO PEI 6329/10 subgenotype 3a standard [accession number AB630970], provided by the Paul Ehrlich Institute, Langen, Germany) and negative (RNase-free water) controls used in the nested RT-PCR were utilized.

### Statistical analyses

HEV seroprevalence and fecal excretion occurrence rates were calculated by dividing the number of animals with HEV antibodies or positive for HEV RNA by the total number tested, with two-sided exact binomial 95% confidence intervals. Statistical analysis, examining factors like age, sex, sampling location, and dates, was conducted using Chi-square or Fisher’s exact test in GraphPad Prism 5.0 software, with significance set at *p* < 0.05.

## Results

A total of five of the 205 rabbits sampled (2.44%; 95% CI: 0.80–5.60) had anti-HEV antibodies (Table 1). The statistical analysis showed no association between the factors analyzed and HEV seroprevalence. Only two collection areas showed seropositive animals, specifically, in Benavente and Noudar.

**Table 1:** Seroprevalence of anti-HEV antibodies in rabbits of Portugal according to region, date of collection, sex and age

Variable	Categories	No. Positives/ no. Analysed	Seroprevalence (%) (95% CI)	*P*-value
Presence of HEV anti-IgG	-	5/205	2.44 (0.80–5.60)	-
Region	Serpa	0/30	0	0.497
	Benavente	3/58	5.17 (1.08–14.38)	
	Alpiarça	0/27	0	
	Mértola	0/5	0	
	Noudar	2/85	2.35 (80.29–8.24)	
Date of collection	2018	1/70	1.43 (0.04–7.70)	0.806
	2019	1/24	4.17 (0.11–21.12)	
	2020	3/151	1.99 (0.41–5.70)	
	2021	0/13	0	
Sex^a^	Female	3/114	2.63 (0.55–7.50)	1.000
	Male	2/89	2.25 (0.27–7.88)	
Age	Adult	4/159	2.52 (0.69–6.32)	1.000
	Juvenile	1/46	2.17 (0.05–11.53)	

^a^Two rabbits were only captured once and due to a field error, their sex was not recorded

The seroprevalence was similar in both adults and juvenile rabbits. Seropositive rabbits were observed in the 2018, 2019 and 2020 sampled years of the study. The proportion of positive sera samples was higher in the sampling year of 2019 (4.17%) than in the years 2018 (1.43%) and 2020 (1.99%). Seroprevalences when observing sex were also similar. From the longitudinally analyzed rabbits only one sample was positive for anti-HEV antibodies. This positive sample belonged to a rabbit that was first sampled (captured) in 2018 and found to be seronegative, and later found seropositive (when deceased) in 2019.

As for HEV detection in feces, HEV RNA was not detected in any of the 120 rabbits (0.0%; 95%CI: 0.00–3.03) by testing with both the broad-spectrum nested and the real-time RT-PCR.

## Discussion

The potential role and impact of rabbits as HEV reservoirs is still in debate worldwide and largely unknown in Portugal. The present study offers the first molecular-based survey of HEV in lagomorphs of Portugal, as well as a longitudinal serological survey.

In recent years, novel strains of HEV have surfaced in various European nations (Bouamra et al. 2014; Abravanel et al. 2017; Oeser et al. 2019; Caballero-Gómez et al. 2019), potentially carrying significant clinical and epidemiological consequences. Furthermore, there has been a growing number of confirmed human cases of HEV-3ra in various European countries, such as France, Belgium, Switzerland, and Spain (Izopet et al. 2012; Abravanel et al. 2017; Suin et al. 2019; Sahli et al. 2019; Rivero-Juarez et al. 2020). The strong similarity observed between HEV-3ra isolates in humans and rabbits provides evidence for suggesting zoonotic transmission associated with the consumption of these animals (Ricci et al. 2017). Moreover, infection with this HEV genotype appears to result in chronic cases of hepatitis E more frequently in immunosuppressed patients, especially compared to other subtypes (Sahli et al. 2019). For these reasons, gaining insights into the involvement of lagomorphs in the epidemiology of HEV is a crucial aspect for managing the rabbit associated emerging genotype. The current study offers fresh epidemiological data on HEV in lagomorph populations of Portugal.

Here a total of five wild rabbits sampled had anti-HEV antibodies, showing further evidence that lagomorphs are naturally exposed to HEV in Portuguese ecosystems, as demonstrated in a previous study (Lopes and Abrantes 2020). Rabbit seroprevalence detected here is lower than in similar studies, like in the United Kingdom (Parisi et al. 2019), Australia (Jenckel et al. 2021), and Portugal (Lopes and Abrantes 2020). Notably, much higher HEV seroprevalences in lagomorphs have been reported in other European countries, such as 30.8% and 37.3% in Germany (Eiden et al. 2016; Hammerschmidt et al. 2017) and 42.9% in Italy (Parisi et al. 2019). Despite the low seroprevalence found in our study on rabbits from Portugal, it is important to approach comparisons between studies cautiously. This involves acknowledging variations in serological methods, study design, species, and the number of animals included in sampling.

Interestingly, HEV seropositivity was detected in 2.52% of adults and 2.17% of juvenile rabbits. While it is possible that yearling individuals may have maternal antibodies, our findings suggest a consistent circulation of HEV within rabbit populations in Portugal. Furthermore, no statistically significant associations were observed among the different age groups. Remarkably, the seropositive animals for anti-HEV antibodies were consistently identified in the regions where the largest number of samples were collected. This intriguing correlation suggests the possibility of a sustained and widespread circulation of HEV among rabbit populations within various ecosystems across Portugal throughout the entire duration of our study.

Although in the present investigation, no statistically significant associations were observed between HEV seroprevalence and factors analyzed, these results indicate a prevalent yet uneven distribution of HEV within rabbit populations of Portugal. Notably, a dually collected adult rabbit showed anti-HEV antibodies in the sample collected post-mortem. The seroconversion suggests HEV may have contributed to the animal’s death, but caution is warranted as it was this rabbit’s death was already linked to hemorrhagic disease virus (RHDV) based on injuries and molecular detection in the liver (Lopes et al. 2023).

Prior investigations have shown the natural occurrence of HEV RNA in lagomorphs, with different prevalence rates (Izopet et al. 2012; Burt et al. 2016). Moreover, studies conducted under experimental conditions have revealed that the HEV RNA in lagomorphs’ feces can be detected within a range of two to ten weeks post-infection (Cheng et al. 2012). In the current investigation, no HEV RNA was detected. These results suggest a restricted occurrence of active HEV infection in rabbits from Portugal. To the best of our knowledge, this is the first study reporting the search for HEV RNA in lagomorphs from Portugal. Moreover, other studies from the neighboring country (Spain) have demonstrated comparable negative findings to ours, but in liver samples (Caballero-Gómez et al. 2020; Castro-Scholten et al. 2023) suggesting a low probability of HEV transmission from wild lagomorphs to other species that coexist with them, including humans.

The current study offers valuable insights, yet it is important to acknowledge its limitations. The sample size of rabbits, although informative, suggests that further studies with possibly larger populations would be beneficial for a more comprehensive understanding. Additionally, the cross-sectional nature of this study offers a snapshot of HEV exposure but does not allow for the determination of causality or the assessment of temporal trends. Also, the reliance on serological tests and PCR for HEV detection, which are standard methods, comes with inherent sensitivity and specificity considerations. Future studies with larger, more diverse samples and longitudinal designs will enhance our understanding of the epidemiology of HEV in rabbit populations. Moreover, although strict measures were taken to maintain RNA quality of isolates (compliance with transport temperatures and use of minimal time for processing samples), RNA degradation can be expected, and no exogenous internal positive control was added to supervise the potential PCR inhibitors.In summary, the serological findings of the present study indicate that rabbits in Portugal underwent natural exposure to HEV, but the viral circulation was limited. The low seroprevalence of anti-HEV antibodies, coupled with the absence of fecal HEV excretion, suggests a limited contribution of these lagomorphs to the HEV epidemiology in Portugal. Additional research across diverse regions of the Iberian Peninsula is essential to attain a more comprehensive and nuanced understanding of the landscape epidemiology of HEV infection in these lagomorph species.

## Data Availability

No datasets were generated or analysed during the current study.
