# Correction: Hepatitis E virus in wild and domestic rabbits from Portugal: a combined molecular and longitudinal serological study

**DOI:** 10.1007/s11259-024-10515-9

**Published:** 2024-08-27

**Authors:** Sérgio Santos-Silva, Nuno Santos, Pedro López-López, Maria S. J. Nascimento, Helena M. R. Gonçalves, Wim H. M. Van der Poel, António Rivero-Juarez, João R. Mesquita

**Affiliations:** 1 School of Medicine and Biomedical Sciences (ICBAS), University of Porto, Porto, Portugal; 2 CIBIO/InBio, Research Center in Biodiversity and Genetic Resources, Campus of Vairão, University of Porto, Vila do Conde, Portugal; 3 Unit of Infectious Diseases, Hospital Universitario Reina Sofia, Clinical Virology and Zoonoses, Instituto Maimonides de Investigación Biomédica de Córdoba (IMIBIC), Universidad de Córdoba (UCO), Cordoba, Spain; 4 Center for Biomedical Research Network (CIBER) in Infectious Diseases, Health Institute Carlos III, Madrid, Spain; 5 Faculty of Pharmacy, University of Porto (FFUP), Porto, Portugal; 6 LAQV, REQUIMTE, Department of Chemistry and Biochemistry, Faculty of Sciences, University of Porto, Porto, Portugal; 7 Quantitative Veterinary Epidemiology, Wageningen University, Wageningen, The Netherlands; 8 Department Virology & Molecular Biology, Wageningen Bioveterinary Research, Lelystad, the Netherlands; 9 Epidemiology Research Unit (EPIUnit), Public Health Institute of the University of Porto, Porto, Portugal; 10 Laboratory for Integrative and Translational Research in Population Health (ITR), Porto, Portugal

**Correction: Veterinary Research Communications**

10.1007/s11259-024-10452-7

In the published version of the article, a typographical error was detected in the introduction section.

The sentence originally reads:

- “Among these, the genus *Paslahepevirus* consists of two species, including *P. balayani*, which comprises HEV genotypes capable of infecting humans and other mammalian species (Nishizawa et al. 2021)*d alci*, infecting moose.”

The correct version should read:

- “Among these, the genus *Paslahepevirus* consists of two species, including *P. balayani*, which comprises HEV genotypes capable of infecting humans and other mammalian species (Nishizawa et al. 2021) and *P. alci*, infecting moose.”

The original article has been corrected.

